# The Neuropeptide PDF Acts Directly on Evening Pacemaker Neurons to Regulate Multiple Features of Circadian Behavior

**DOI:** 10.1371/journal.pbio.1000154

**Published:** 2009-07-21

**Authors:** Bridget C. Lear, Luoying Zhang, Ravi Allada

**Affiliations:** Department of Neurobiology and Physiology, Northwestern University, Evanston, Illinois, United States of America; Howard Hughes Medical Institute/Stanford University, United States of America

## Abstract

Discrete clusters of circadian clock neurons temporally organize daily behaviors such as sleep and wake. In *Drosophila*, a network of just 150 neurons drives two peaks of timed activity in the morning and evening. A subset of these neurons expresses the neuropeptide pigment dispersing factor (PDF), which is important for promoting morning behavior as well as maintaining robust free-running rhythmicity in constant conditions. Yet, how PDF acts on downstream circuits to mediate rhythmic behavior is unknown. Using circuit-directed rescue of PDF receptor mutants, we show that PDF targeting of just ∼30 non-PDF evening circadian neurons is sufficient to drive morning behavior. This function is not accompanied by large changes in core molecular oscillators in light-dark, indicating that PDF RECEPTOR likely regulates the output of these cells under these conditions. We find that PDF also acts on this focused set of non-PDF neurons to regulate both evening activity phase and period length, consistent with modest resetting effects on core oscillators. PDF likely acts on more distributed pacemaker neuron targets, including the PDF neurons themselves, to regulate rhythmic strength. Here we reveal defining features of the circuit-diagram for PDF peptide function in circadian behavior, revealing the direct neuronal targets of PDF as well as its behavioral functions at those sites. These studies define a key direct output circuit sufficient for multiple PDF dependent behaviors.

## Abstract 2

Animals use distinct sets of clock neurons to time behaviors in the morning and evening. In this article, the direct neural targets for morning neurons and the neuropeptide pigment dispersing factor are revealed in the fruit fly.

## Author Summary

Animals depend on being awake at the right time of day to find food and mates and fend off predators. Circadian pacemaker neurons in the brain play a crucial role in timing of specific behaviors to the appropriate times of day. These neurons are further specialized to those primarily responsible for morning and evening behavior. We have used the fruit fly *Drosophila* as a simple model system to elucidate the neural circuits important for timed daily behavior. In flies, a small group of clock neurons devoted to morning behavior express a neuropeptide, PIGMENT DISPERSING FACTOR (PDF). Until now it was unclear what the direct neural targets of this peptide are and how its actions at those targets mediate timed behavior. Here we find that the so-called morning clock neurons communicate directly to other clock neurons, those responsible for evening behavior. This communication sustains high amplitude morning activity and sets the phase of evening activity as well as the period of activity rhythms in constant conditions. These studies reveal the circuit diagram for PDF function in circadian behavior.

## Introduction

Circadian clocks act in many organisms to promote daily rhythms of behavior and physiology. In *Drosophila*, clock function under conditions of light-dark entrainment (12-h light∶12-h dark; LD) is evident as increases in locomotor activity in advance of lights-on (morning anticipation) and lights-off (evening anticipation). These rhythms are driven by well-conserved transcriptional feedback loops in which the basic helix-loop-helix transcription factor heterodimer, CLOCK/CYCLE, activates components such as *period* (*per*), *timeless* (*tim*), and *clockwork orange* (*cwo*) that feedback and regulate CLOCK/CYCLE binding to its cognate DNA targets [1]–[4]. These feedback loops generate daily gene expression rhythms.

Approximately 150 pacemaker neurons in the adult *Drosophila* brain are implicated in the regulation of circadian locomotor behavior. These neurons can be roughly divided into the PIGMENT DISPERSING FACTOR (PDF)-expressing small and large LNv (sLNv, lLNv), a single non-PDF sLNv, the dorsal lateral neurons (LNd), and three groups of dorsal neurons (DN1, DN2, and DN3) [5]. Ablation of PDF+ neurons results in substantial reduction in morning anticipation [6],[7]. A functional clock in the small subset of PDF+ neurons is sufficient to drive morning behavior, and these cells have thus been dubbed “morning” (M) cells [8]. The large LNv have been observed to promote arousal especially during the light period [9]–[11]. A subset of ∼30 circadian pacemaker neurons, including the non-PDF sLNv, LNd, and/or a small subset of DN1s and DN3s [7],[8],[12],[13], are essential for evening anticipatory behavior, and are thus dubbed “evening” (E) cells. Mammalian circadian clocks may also have a similar morning and evening organization [14],[15].

*Drosophila* also maintains robust locomotor activity rhythms during constant-dark conditions (DD), reflecting the endogenous function of its circadian clock. The PDF-expressing LNv play a critical role in sustaining free-running rhythms, as ablation of the PDF+ LNv leads to decreased DD rhythmicity [6]. Moreover, tissue-specific rescue experiments indicate that the circadian clock component PERIOD (PER) [7] and the circadian output ion channel NARROW ABDOMEN (NA) [16] are each required in the PDF+ LNv to promote robust, sustained DD rhythmicity. The function of PDF neurons is instructive, as selectively altering the period of these cells drives changes in period length in several non-PDF neurons and sets the circadian period of locomotor activity [17]. It is not known if the ability of PDF neurons to influence non-PDF pacemaker neurons reflects a direct cellular connection.

The PDF neuropeptide is implicated as the principal transmitter of the LNv group, as flies lacking *Pdf* function exhibit phenotypes similar to ablation of the PDF+ LNv [6]. In LD, these phenotypes include reduced morning behavior and advanced evening behavior. During DD, null *Pdf^01^* mutants exhibit progressive dampening of locomotor rhythmicity and a slightly shortened period. A receptor for *Drosophila* PDF has been identified (*PDFR*, aka *han*, *groom-of-pdf*, *CG13758*), and loss of this receptor leads to circadian phenotypes essentially identical to *Pdf^01^* mutants [18]–[20].

The DD behavioral phenotypes of *Pdf^01^* mutants are accompanied by alterations in the molecular clock. PER oscillations in the DN1 of *Pdf^01^* mutants rapidly damp during DD, indicating a role for PDF in sustaining molecular rhythms [21]. In contrast, the LNd of *Pdf^01^* mutants exhibit persistent rhythms, but with an advance in the phase of PER oscillations, consistent with the observed short behavioral period of these flies [22]. Additionally, desynchronized PER nuclear localization rhythms are observed in the sLNv of *Pdf^01^* mutants, but only after many days of DD [22]. These data suggest that PDF may also reset or synchronize these molecular clocks. However, molecular alterations have not been observed in *Pdf^01^* mutants in LD [23], suggesting that PDF may be acting downstream of the molecular clock under these conditions.

While the molecular consequences of manipulating PDF/PDF RECEPTOR (PDFR) function have been well described, it was not previously known which of these effects reflected the direct actions of PDF on the affected cells or whether they were mediated by cellular intermediates. In addition, it was not known which of these direct cellular targets was mediating the multiple effects of PDF on behavior, especially under LD conditions. Here we demonstrate that PDFR expression limited to the ∼30 non-PDF evening cells can not only alter the timing of evening behavior, but also drive the amplitude of morning behavior. Our data indicate that the effect of PDFR expression on morning behavior does not likely occur through the core clock, but instead through the regulation of neuronal output. We also demonstrate a role for PDFR in non-PDF cells to reset evening phase and regulate period length, consistent with core clock resetting. Finally, we find that PDFR likely functions within a more distributed group of pacemaker neurons, including the PDF+ LNv, to promote sustained DD rhythmicity. This study defines the major direct targets for PDF in vivo and their functions in circadian behavior.

## Results

### PDFR Expression Restricted to Non-PDF Evening Cells Rescues Both Evening Phase and Morning Behavior

**Figure 1:**
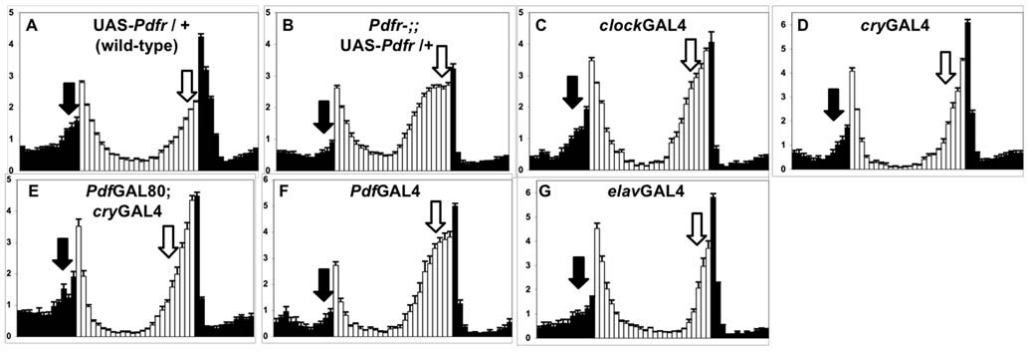
Expression of PDFR in evening cells rescues both morning and evening anticipation in *Pdfr* mutants. (A–G) Normalized activity plots for adult male populations, averaged over 4 d of LD entrainment. Light phase is indicated by white bars, while dark phase is indicated by black bars. Error bars represent standard error of the mean (*n* = 26–82). Arrows indicate morning anticipation (black) and evening anticipation (white). (A) UAS-*Pdfr*/+; (B–G) *Pdfr^han5304^*; UAS-*Pdfr*/+ with the following heterozygous GAL4 and GAL80 drivers; (B) None; (C) *clock*GAL4; (D) *cry*GAL4-13; (E) *Pdf*GAL80; *cry*GAL4-13; (F) *Pdf*GAL4; (G) *elav*GAL4 (second chromosome).

To define the neuroanatomical targets of PDF action in circadian behavior, we performed tissue-specific rescue of a *Pdfr* mutant using the GAL4-UAS system. For these experiments, we utilized a strong loss-of-function mutant allele of *Pdfr*, *han5304*. Like null *Pdf^01^* mutants, *Pdfr ^han5304^* flies display strongly reduced morning anticipation and phase-advanced evening anticipation in LD, as well as a reduced morning peak at the onset of DD [6],[18]. Previous studies had suggested that PDFR functions in circadian neurons largely based on partial rescue using a single *per*GAL4 driver [18]. *per*GAL4 drivers, in addition to demonstrating expression in all major circadian pacemaker groups, also drive widespread expression in nominally noncircadian brain areas, including the central complex, antennal lobe, and lateral horn [24], raising questions as to the precise site of PDFR function. To address this issue, we utilized *clock*GAL4 [25], which drives broad expression among all major circadian neuronal groups [16] but relatively limited noncircadian expression, including the pars intercerebralis (PI) and cells surrounding circadian neurons [16]. Using this driver, we find that PDFR expression in *Pdfr* mutants rescues morning anticipation and the proper timing of LD evening behavior (Figures 1A–1C and S1; *p*<0.05). Given the relatively limited noncircadian expression of *clock*GAL4, these results suggest a major function for PDFR in circadian neurons.

**Figure 2:**
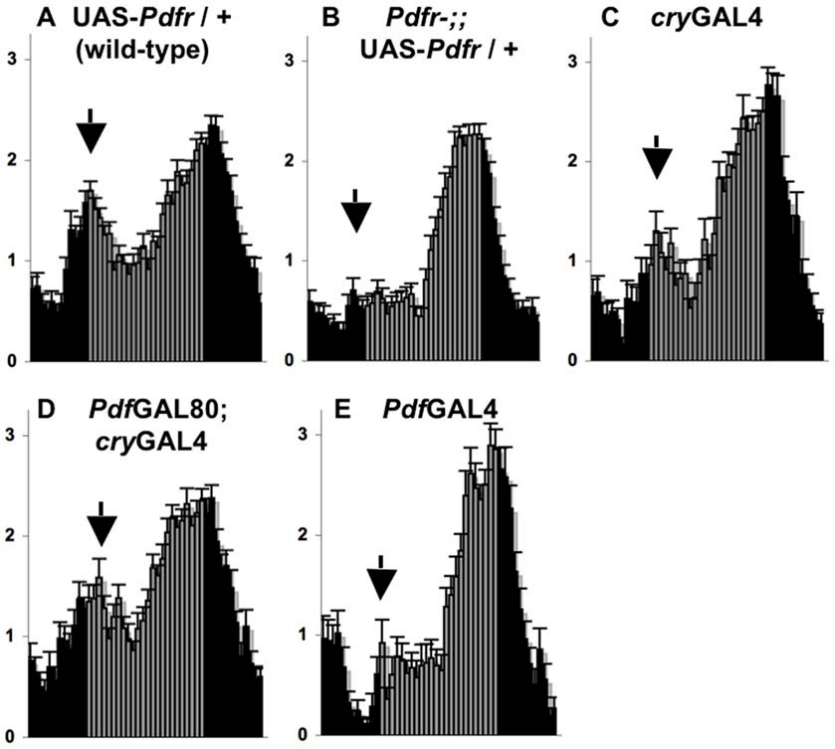
Expression of PDFR in evening cells rescues morning behavior on the first day of constant darkness. (A–E) Normalized activity plots of adult male populations over the last 6 h of LD (ZT 18- CT0) followed by the first 18 h of DD (CT 0-18). Presumptive light phase (CT 0-12) is indicated by gray bars. Error bars represent standard error of the mean (*n* = 30–76). Arrows indicate morning behavior. (A) UAS-*Pdfr*/+; (B–E) *Pdfr^han5304^*; UAS-*Pdfr*/+ with the following heterozygous GAL4 and GAL80 drivers: (B) none; (C) *cry*GAL4-13; (D) *Pdf*GAL80; *cry*GAL4-13; (E) *Pdf*GAL4.

We next assessed PDFR function specifically in the pacemaker neuron subsets known to control morning and evening behavior. We performed rescue using a GAL4 driver containing the promoter and first intron of the *cryptochrome* gene (*cry*GAL4-13) [26]. *cry*GAL4-13 drives expression in both PDF-expressing morning cells and ∼30 non-PDF evening cells (LNv, LNd, small subset of DN1 and DN3), while promoting little or no expression in other circadian pacemaker neurons (e.g., most DN1, all DN2, and most DN3) or outside the circadian system [7],[13],[16]. *cry*GAL4-13 driven expression of UAS-*Pdfr* restores the timing of evening behavior (*p*<0.0001) and also promotes significant restoration of morning behavior during LD and the first day of DD (DD1; Figures 1D, 2A–2C; Table 1; *p*<0.001). We examined and quantified morning behavior during DD1 as the lights-on response in LD can mask some of the clock-driven morning behavior. We then further restricted UAS-*Pdfr* expression specifically to either the evening cells or morning cells. Expression was restricted to evening cells by blocking GAL4 induction selectively in PDF+ cells using GAL80 (*Pdf*GAL80; *cry*GAL4-13), while morning cell-specific expression was driven using *Pdf*GAL4. Expressing UAS-*Pdfr* only in non-PDF *evening* cells rescues both the timing of evening behavior and the magnitude of morning anticipation (Figures 1E and 2D; Table 1; *p*<0.0001). In contrast, UAS-*Pdfr* expression restricted to morning cells does not have comparable effects on morning or evening behaviors (Figures 1F and 2E; Table 1), as previously reported [18]. These findings suggest that the PDF+ LNv can communicate directly to the non-PDF “evening” cells through PDFR to promote morning behavior.

**Table 1:** Rescue of morning and evening *Pdfr* phenotypes.

Genotype	Time of Evening Anticipation	*n*	DD Day 1 Morning Index	*n*
UAS-*Pdfr*/+	10.4±0.2	58	0.8±0.1	56
*Pdfr^han5304^*;;UAS-*Pdfr*	8.6±0.1	82	0.2±0.0	76
*cry*GAL4-13	11.4±0.1	44	0.6±0.1	44
*Pdf*GAL80;*cry*GAL4-13	11.2±0.2	51	0.7±0.1	50
*Pdf*GAL4	9.3±0.2	30	0.3±0.1	30

### PDFR Likely Functions in Evening Cells to Promote Morning Behavior through an Effect on Circadian Output

We next examined whether the behavioral contribution of evening cells to morning behavior might be driven by changes in the circadian clock. The etiology of circadian phenotypes in flies with disrupted PDF signaling has largely focused on the role of PDF in synchronizing and/or resetting circadian clocks. These studies have largely identified changes in molecular oscillations of core clock components, such as PER, which reflect core clock timing, under constant darkness conditions. It has been proposed that light can compensate for the loss of PDF/PDFR as no large changes in the core clock have been described in *Pdf^01^* mutants in LD [23]. However, these experiments were performed with only two time points.

**Figure 3:**
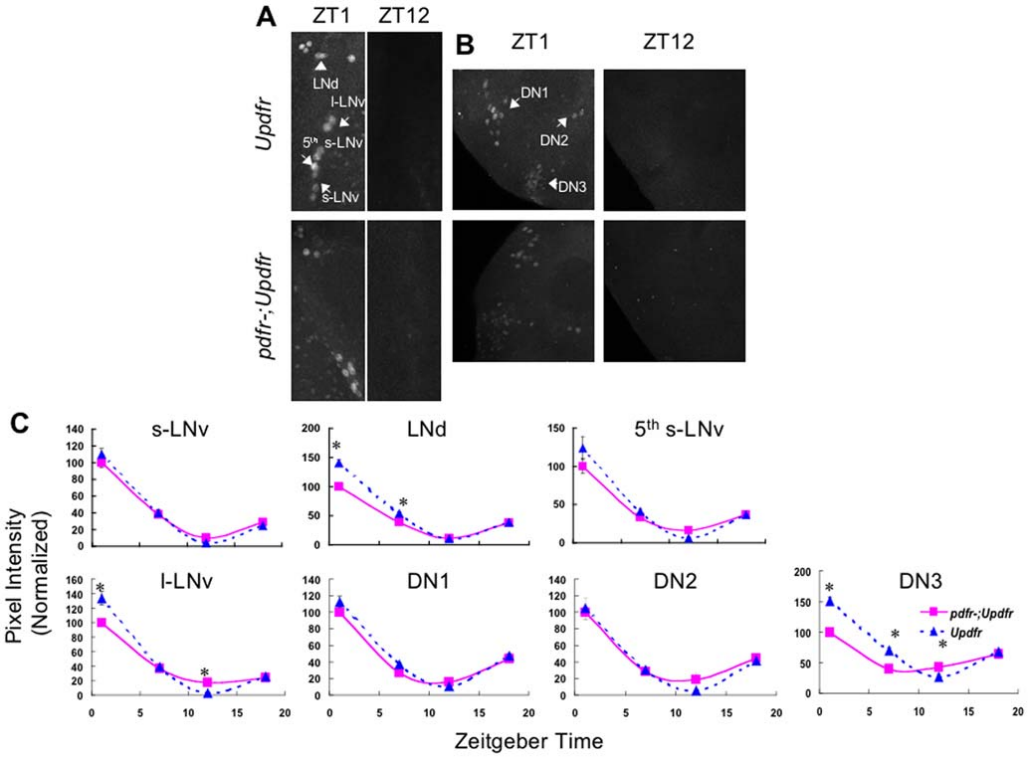
PERIOD cycles robustly in *Pdfr* mutants during LD. (A,B) Maximum projections of confocal sections taken in representative adult UAS-*Pdfr* and *Pdfr^han5304^*;UAS-*Pdfr* brains labeled with PER antibody. Sections contain either the lateral neurons (A) or the dorsal neurons (B) at ZT1 and ZT12, which are times of peak and trough PER expression. LN and DN subgroups are indicated by arrows. (C) Plots of average normalized pixel intensity versus Zeitgeber time for each pacemaker cell group for UAS-*Pdfr/+* (dashed blue line) and *Pdfr^han5304^*; UAS-*Pdfr/+* (solid pink line). See Materials and Methods for details of quantification method. Error bars represent standard error of mean. The results are a combination of two independent experiments: s-LNv, *n* = 32–54; LNd, *n* = 28–88; 5th s-LNv, *n* = 10–16; DN1, *n* = 50–198; DN2, *n* = 11–24; DN3, *n* = 73–316. Only ZT1 and ZT7 for the LNd and ZT1, 7, and 12 for DN3 are statistically different from controls (*p*<0.001).

Given our interest in determining the molecular basis of morning and evening behavioral phenotypes in LD, we performed PER immunolabeling in wild-type (*UAS-Pdfr/+*) and *Pdfr* mutant (*Pdfr ^han5304^*; *UAS-Pdfr/+*) flies during LD using four time points. Previous studies have shown that PER expression restricted to PDF neurons is sufficient to rescue morning anticipation of *per^01^* mutants, suggesting that PDF actions do not require clock function in non-PDF neurons for morning behavior [8]. We asked whether changes in the LNv clock could account for loss of morning behavior in *Pdfr* mutants. However, no significant differences in PER oscillations were observed in the PDF+ sLNv important for morning behavior (Figure 3A–3C). While small changes were observed in some pacemaker neuron clusters (lLNv, LNd, and DN3), high amplitude oscillations were observed in all pacemaker neurons groups in *Pdfr* mutants, in contrast to the highly significant reduction in morning behavior (Figure 2). In addition, clock function in the lLNv is not required for morning behavior [7],[8],[27], suggesting (but not excluding) the possibility that these small molecular changes do not underlie changes in morning behavior. The LNd and a subset of DN3 have been implicated in regulating evening behavior [7],[13], and subtle changes in the LNd and/or DN3 may be responsible for the ∼2-h phase advance in evening anticipation (Figure 1B). A higher temporal resolution will be necessary to definitively demonstrate a molecular phase shift in these cell clusters. Nonetheless, relatively small phase changes are unlikely to explain large amplitude changes in morning behavior. In this case, the dramatic effects of PDFR on morning behavior largely reflect its function in circadian output.

### PDFR Function in a Distributed Network Including PDF Cells Contributes to DD Rhythms

In addition to defects in morning and evening behaviors, *Pdf^01^* and *Pdfr* mutants exhibit decreased rhythmic power and shortened period length in DD [6],[18],[19]. To determine whether the anatomical requirements for PDFR function in free-running rhythmicity match those for morning and evening behaviors, we assessed DD rhythms in PDFR rescue flies. Expression of PDFR using a broad circadian driver *clock*GAL4 promotes significant rescue of DD rhythmicity, as reflected by rhythmic power (*p*<0.0001) and period length (*p*<0.0001; Table 2). Period length of *clock*GAL4 rescue flies is slightly short (23.3+/−0.1 h), yet comparable to *clock*GAL4 driven overexpression of PDFR in a wild-type background (23.4+/−0.1 h; Table 2), likely due to a modest (∼30 min) overexpression effect (Table 2). Nonetheless, *clock*GAL4 rescue of period is statistically significant and supports a role for PDFR in circadian neurons to promote normal DD period and rhythmicity.

**Table 2:** Rescue of free-running period length and rhythmicity.

Genotype	Period (h)	Power	Percent Rhythmic	*n*
Rescue				
*Pdfr^han5304^*;;UAS-*Pdfr/+*	22.9±0.1	25±2	61	134
*Pdfr^han5304^;PdfGAL4/+*; UAS-*Pdfr/+*	23.0±0.1	24±5	60	30
*Pdfr^han5304^;;cryGAL4-13*/UAS-*Pdfr*	23.9±0.1	55±6	91	43
*Pdfr^han5304^;Pdf*GAL80/+; *cry*GAL4-13/UAS-*Pdfr*	23.7±0.1	29±4	76	49
*Pdfr^han5304^;;clockGAL4*/UAS-*Pdfr*	23.3±0.1	68±6	93	44
*Pdfr^han5304^;elavGAL4/+*	23.2±0.1^a^	10±3	24	38
*Pdfr^han5304^;elavGAL4/+*; UAS-*Pdfr/+*	23.9±0.0	147±6	100	53
*Pdfr^han5304^;elavGAL4/PdfGAL80*; UAS-*Pdfr/+*	23.9±0.1	66±8	83	30
*PdfrGAL4/Pdfr^han5304^*	23.1±0.2	21±4	43	40
*PdfrGAL4/Pdfr^han5304^*;; UAS-*Pdfr/+*	23.7±0.1	94±7	100	40
Overexpression				
UAS-*Pdfr/+*	23.9±0.0	76±5	100	56
*PdfGAL4/+*	24.1±0.1	86±10	93	14
*PdfGAL4/+*; UAS-*Pdfr/+*	24.1±0.0	90±10	90	29
*cryGAL4-13*/UAS-*Pdfr*	23.9±0.0	81±8	100	25
*PdfGAL80/+*; *cryGAL4-13*/UAS-*Pdfr*	23.6±0.1	76±9	96	26
*clockGAL4/+*	23.9±0.1	68±10	79	24
*clockGAL4*/UAS-*Pdfr*	23.4±0.1	82±7	97	30
*elavGAL4/+*; UAS-*Pdfr/+*	23.9±0.1	113±10	96	23

a Single weakly rhythmic 31-h fly not included in period calculation.

To assess PDFR DD function in specific circadian neuron subsets, we analyzed DD rescue using GAL4 drivers with limited circadian expression. Expression of PDFR in morning and evening cells using *cry*GAL4-13 rescues DD period length and partially restores DD rhythmicity (Table 2; *p*<0.0001), suggesting broader or stronger DN expression provided in *clock*GAL4 may be needed for robust rhythmicity. However, further restriction of PDFR expression to evening cells using *Pdf*GAL80 fully blocks the *cry*GAL4-13 rescue of rhythmic power (*p* = 0.6; Table 2). While we cannot rule out residual GAL4 activity, these data are consistent with full GAL80 repression of GAL4 activity in the LNv. These data uncouple LD and DD rescue and suggest a role for PDFR within the PDF+ LNv to promote DD rhythms. Yet consistent with previous findings [18], PDFR expression restricted to PDF+ neurons (*Pdf*GAL4) has no significant effect on either free-running rhythmicity or period length in DD, indicating that PDFR function in these cells is not sufficient for normal DD rhythms (Table 2). To confirm a role for PDFR in the PDF+ neurons, we expressed *Pdf*GAL80 in the context of pan-neuronal *elav*GAL4-mediated rescue. *elav*GAL4 expression results in strong rescue of all LD and DD phenotypes (Figure 1G, Table 2; *p*<0.0001). As with *cry*GAL4-13, blocking *elav*GAL4 driven PDFR selectively in the PDF+ LNv using *Pdf*GAL80 results in a substantial reduction in rhythmic power (Table 2; *p*<0.0001). Taken together, these data suggest that PDF/PDFR communication within the LNv plays an important role in sustaining robust DD rhythmicity. In addition, our rescue also suggests that other cells also contribute to DD rhythmicity.

**Figure 4:**
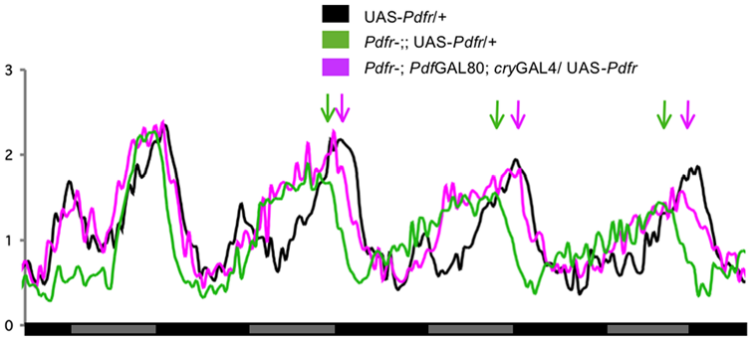
Expression of PDFR in evening cells rescues free-running period length. Normalized activity plots of adult male populations over the last 6 h of LD followed by 5 d of DD. Genotypes are indicated. *n* = 50–76. Arrows indicate phase difference between *Pdfr* mutant (green) and rescue flies (pink).

Notably, period length is also significantly rescued with all GAL4 drivers tested except *Pdf*GAL4 (Table 2; *p*<0.0001). Yet unlike rhythmic power, period length rescue is unchanged when PDFR expression is blocked in PDF+ neurons via *Pdf*GAL80. In fact, PDFR expression restricted only to evening cells (*Pdf*GAL80; *cry*GAL4-13) retains significant rescue of period length, as evident from group activity profiles (Figure 4) and individual fly analyses (Table 2). This remains true even if only strongly rhythmic flies (Power≥40) are considered (unpublished data; *p*<0.0001). Thus, direct PDF communication among PDF-expressing neurons, as well as with other target neurons, is important for sustaining DD rhythms. In contrast, the PDF+ LNv communicate directly to non-PDF evening cells to set period length, indicating functional and anatomical specialization of PDF signaling.

### Endogenous *Pdfr* Regulatory Sequence Drives Expression in Specific Circadian Neurons as Well as Noncircadian Expression in the Adult *Drosophila* Brain

Taken together, our functional neuroanatomical approaches highlight PDFR function in circadian pacemaker neurons. However, reports of the PDFR expression pattern are conflicting. Two initial reports utilized independently generated antisera to assess PDFR expression in the *Drosophila* brain. One reported expression limited mainly to circadian neurons [18], while the other observed broad expression that included only few circadian neurons [20]. We previously reported *pdfr* expression using in situ hybridization and noted expression in potential dorsal neurons and the PI [19]. A more recent report indicates that the reported immunofluorescence patterns may not represent specific PDFR signal [28], calling into question the true PDFR expression pattern. We have made several additional attempts to generate specific antisera to PDFR but have yet to identify reproducible and robust signals (unpublished data).

To examine *Pdfr* expression, we instead used a P-element exchange strategy to insert a P{GAL4} element ∼40 bp upstream of the presumptive *Pdfr* transcription start site (*Pdfr*GAL4; see Materials and Methods) [29]. A targeted GAL4 insertion into a locus of interest has been a valuable approach to report endogenous gene expression patterns [30]. If the GAL4 insertion falls under the control of enhancers that normally drive *Pdfr* expression, we predict that *Pdfr*GAL4 will reflect endogenous *Pdfr* expression. In this case, *Pdfr*GAL4 if combined with UAS-*Pdfr* should be able to rescue *Pdfr* mutant phenotypes. The original insert used to generate *Pdfr*GAL4 displayed a modest circadian rhythmicity phenotype [20] and a ∼50% reduction in transcript levels (unpublished data). Consistent with these data, we find that *Pdfr*GAL4/*Pdfr^han5304^* flies display poor DD rhythmicity (Table 2). Importantly, this reduced rhythmicity is strongly rescued by *Pdfr*GAL4 driven expression of UAS-*Pdfr* (Table 2; *p*<0.0001), suggesting that *Pdfr*GAL4 is a faithful reporter of *Pdfr* expression.

**Figure 5:**
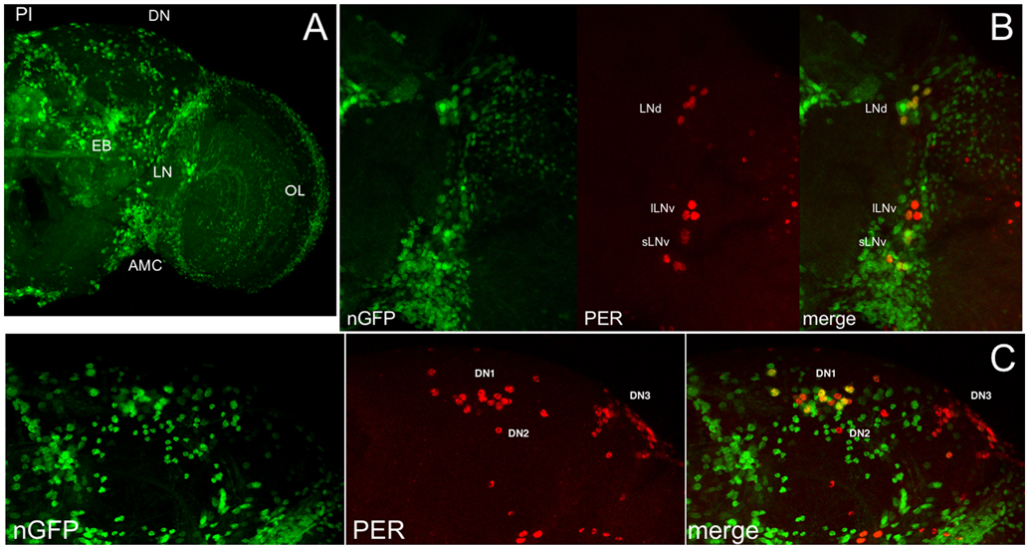
*Pdfr*GAL4 drives broad expression in the adult brain that includes circadian neurons. (A–C) Maximum projections of confocal sections from *Pdfr*GAL4/+;; UAS-nGFP/+ adult brains. (A) nGFP signal in a representative brain hemisphere. GFP-expressing cell groups include the PI, ellipsoid body (EB), optic lobe (OL), antennomechanosensory center (AMC), and brain regions containing circadian lateral neurons (LN) and dorsal neurons (DN). (B,C) *Pdfr*GAL4/+;; UAS-nGFP/+ brains are labeled with antibodies to PER. Examination of the nGFP (green), PER (red), and merged images indicates that *Pdfr*GAL4 drives expression in several LNv and LNd (B), as well as several DN1 (C).

We then examined the driven expression pattern for *Pdfr*GAL4. Upon crossing *Pdfr*GAL4 to UAS-nuclear green fluorescent protein (GFP) (nGFP), we observe broad GFP expression in the adult *Drosophila* brain, including circadian neuron regions, PI, optic lobe, and ellipsoid body (Figure 5A), the latter possibly consistent with noncircadian functions of PDF in arousal and geotaxis [9]–[11],[20],[31]. Given our rescue data, we more closely examined expression within circadian pacemaker neurons. To directly assess circadian expression, we labeled *Pdfr*GAL4 UAS-nGFP brains with PER antisera. We observe prominent GFP expression in the sLNv, all LNd, and several DN1 (Figure 5B and 5C). The PI and DN expression is consistent with our published in situ expression pattern [19]. Weak expression is observed in the lLNv. We also consistently observe expression in two DN3s, and we sometimes observe expression in one of the two DN2s (unpublished data). These expression data nicely complement our functional neuroanatomy data. Expression in the sLNv is consistent with a role for PDFR in these cells to sustain free-running rhythmicity (see *Pdf*GAL80), as the sLNv are known to be especially important for DD rhythmicity [8],[17]. *Pdfr* expression in the LNd, DN1, and DN3 subset is consistent with our data demonstrating an important role of the non-PDF evening cells in morning and evening behavior and DD period length.

## Discussion

Here we define the direct targets of PDF using circuit-specific rescue and find that the direct action of PDF on just ∼30 neurons, the so-called evening pacemaker neurons, mediates PDF dependent effects on morning, evening, and free-running behaviors. We corroborate our functional rescue data with a novel GAL4 enhancer trap reporting endogenous *pdfr* expression. We also provide strong evidence that PDF, in addition to its well-described effects on the core clock mechanism, also likely affects the output of pacemaker neurons providing novel mechanistic insight into PDFR function. These studies define a major direct conduit for in vivo PDF signaling in circadian behavior.

A number of reports have examined the molecular consequences of manipulating PDF neuron function. Altering the core clock, output, or projections of PDF neurons alters the molecular clock in non-PDF circadian neurons and evening behavior under short days or in constant darkness [17],[19],[22],[23],[32]–[36]. However, these studies leave open a number of key questions important for elucidating the PDF circuit diagram. Not surprisingly, functional changes in PDF neurons can be propagated widely through the nervous system, not only to the direct cellular targets of that group of neurons (primary target neurons), but to the targets of those targets (secondary), and so on (tertiary). Thus, the direct and indirect effects of PDF could not be distinguished in these papers. In addition, these studies do not identify the behavioral functions of PDFR (or PDF) at these different cellular targets particularly on LD morning and evening behavior. Some of these studies also rely on analysis of mutant flies with significant developmental abnormalities [34],[36].

**Figure 6:**
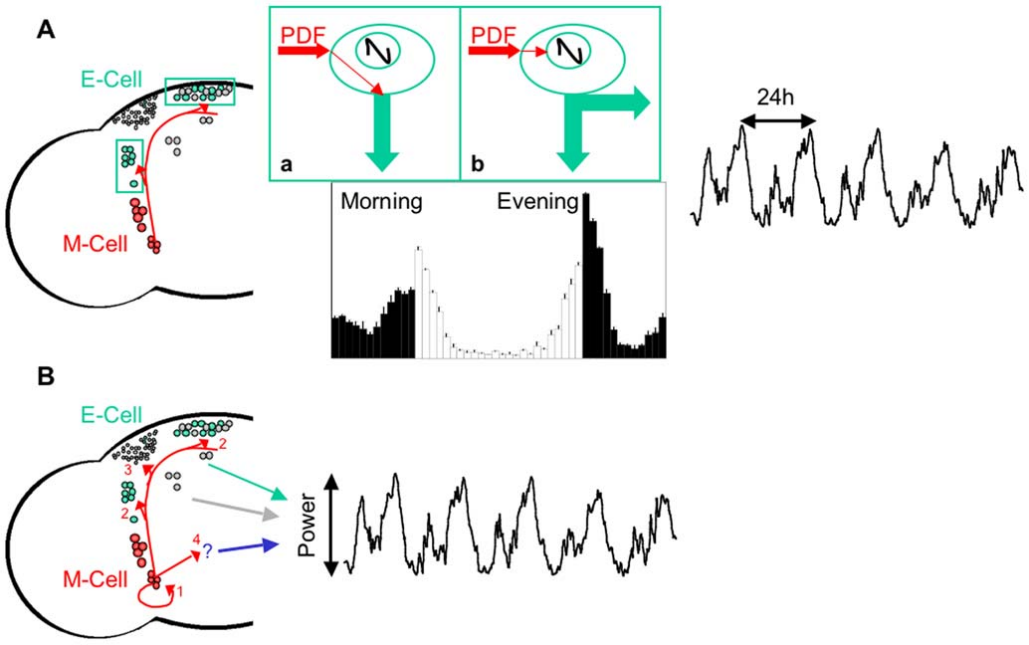
Neuronal circuit diagram for PDF-mediated circadian locomotor behavior. (A) In LD, M-cells (red), including PDF+ l- and s-LNvs, signal to E-cells (green, including PDF-5th s-LNv, the LNds, several of the DN1s, and two DN3s) via PDF. E-cells in turn drive morning anticipation and set the phase of evening anticipation. White and black bars indicate activities in the light and dark phase, respectively. In DD, M-cells employ PDF to communicate to E-cells, which determines the length of behavioral period. A wild-type DD activity profile is shown in black. Insets (a) and (b) demonstrate how PDF signaling from M-cells regulates circadian behavior via E-cells. (a) In LD, PDF signaling likely directly regulates an output pathway of E-cells, which drives morning anticipation. (b) PDF signaling regulates E-cell clock, which sets the phase of evening anticipation in LD and the length of behavioral period in DD likely by resetting core oscillators. Black curve represents the core molecular oscillator. Red arrows indicate PDF signaling inputs, whereas green arrows indicate an output pathway of the E-cells. (B) In DD, PDF+ s-LNvs employ PDF to communicate among (1) themselves (2) E-cells, (3) other circadian neurons (gray, including several of the DN1s, most of the DN3s, and the LPNs), and (4) noncircadian regions that remain to be identified, which all may contribute to maintenance of robust behavioral rhythms. Red arrows indicate PDF signaling to different anatomical targets. Green, gray, and blue arrows indicate output pathways from E-cells, other circadian neurons, and noncircadian regions, respectively.

Measurements of PDF activation in ex vivo brains have also been used to infer direct cellular targets [28]. Bath application of PDF to cultured brains up-regulated cAMP levels. However, these assays required ∼1 min to observe significant activation. Given the slow response time course relative to the faster rate of synaptic transmission, PDF effects on a primary target neuron could be propagated through circuitry to secondary neurons to increase cAMP, on a similar minute time course. Thus, one cannot exclude the possibility that some of the observed responses may be indirect. In addition, effects might even reflect direct responses to nonphysiological levels of PDF. Moreover, this study does not address the behavioral functions of PDF at those sites. By using the direct molecular target of PDF, the PDF receptor, to rescue *Pdfr* mutant phenotypes, we functionally define these direct neuronal targets in vivo. We demonstrate that the expression of PDFR in a highly restricted group of neurons (∼30 neurons) is sufficient to rescue morning behavior, evening phase, and circadian period, thus defining a major direct output circuit for multiple PDF-dependent behaviors (Figure 6).

How does PDF function at these neuronal targets? Previous studies have identified molecular clock changes especially under constant darkness conditions indicating that PDF acts to reset molecular oscillators. Consistent with this model, we observed that PDFR expression in the E cells can rescue circadian period and evening activity phase. Moreover, we identified subtle molecular changes in E cells in LD that are consistent with a small phase shift in molecular oscillations. Thus, at least some PDF-dependent behaviors can be attributed to its function in resetting clocks.

While there are PDF effects on core clocks, our data also suggest an additional output function particularly in regulating morning behavior (Figure 6). Both *Pdf* and *Pdfr* mutants have been shown to have strong effects on the amplitude of morning behavior. Our studies similarly demonstrate major changes in the amplitude of morning behavior despite robust oscillations in both the sLNv (which are sufficient for morning behavior) as well as other circadian cell groups including the E cells. The published data further support this model. The finding that PDF can acutely affect neuronal firing rate in other insects [37] strongly suggested that such clock-independent output functions were possible, if not likely, as core clock changes and their subsequent translation into neuronal firing changes would take place over a longer time frame. It has previously been shown that rescue of clocks exclusively in the PDF neurons in the arrhythmic *per^01^* mutant rescues morning behavior [8]. If morning behavior works by PDF targeting of the E cells, then PDF must act on the output of the E cells in these flies, as there is no clock in the E cells. In addition, manipulation of sodium channel activity shifts the phase of PDF rhythms and morning behavior but these are not accompanied by shifts in molecular oscillators in the sLNv, LNd, or DN1, consistent with an output function [33]. Taken together, we believe our data coupled to the published literature support the notion that PDF can affect the output of E cells in addition to its phase resetting effects.

Previous data have suggested that PDF activates MAPK phosphorylation in the dorsal brain just prior to the increase in morning activity [38], suggesting that PDF release may promote morning activity. Consistent with this hypothesis, recent data suggest a role for the PDF expressing large LNv in driving locomotor activity and arousal [9]–[11],[39]. These effects may be mediated through the sLNv, which in turn project to the dorsal brain [10],[11]. PDF release in the morning may also reset oscillators in the E cells (Figure 6).

Our identification of a role for so-called “E” cells in M behavior, also fits well with prior data suggesting that E cells can control M behavior and highlights additional complexity of the M-E model. Manipulating the clock in E cells can shift morning behavioral phase under long photoperiods [40], whereas rescue of the arrhythmic *per^0^* mutant in non-PDF neurons can rescue morning behavior [7]. However, these results were interpreted to indicate that E cell clocks signal through M cell clocks to drive morning behavior. Indeed, these authors proposed that M cells signal through unknown circuits to drive morning behavior [7]. Here we demonstrate that the E cells themselves are direct targets of the M cells to drive morning behavior. Given our data, E cells may signal to other pacemaker neurons or even nonpacemaker neurons rather than to M cells to drive morning behavior.

How then does one reconcile the apparent observation that clock function is sufficient in E cells to drive morning behavior with the observation that E cells are not necessary for M behavior [7]? One possibility is that redundant pathways control morning behavior. Thus, PDF communication to E cells is sufficient, but may not be necessary, to drive morning behavior. Nonetheless, these data demonstrate that the function of M and E cells is more intertwined than previously thought, necessitating a revision of the simplest versions of the M-E model.

As E cells constitute a focused yet heterogeneous group of cells [7],[13],[35],[41],[42], it will be of interest to determine whether distinct subsets of them are responsible for E and M behavior. E cells consist of the non-PDF small LNv, two DN3s, the LNd, which can be further subdivided by their expression of Neuropeptide F (NPF) [43], and a subset of DN1s, two of which persist from larval development and the remainder that express the transcription factor GLASS [21]. We have attempted to rescue *Pdfr* mutant phenotypes using *Pdf*GAL4 and *npf*GAL4 combined, but we fail to observe significant rescue of any LD or DD phenotypes (unpublished data), suggesting a role for PDFR in E cells other than the NPF-expressing LNd. The DD period is likely driven from some or all LNd, as DN1 rhythms of *Pdf^01^* mutants rapidly damp in DD while LNd rhythm persist with a short period for several days in DD [22], comparable to the period of DD locomotor rhythmicity in *Pdf^01^* or *Pdfr* mutants. Moreover, since the residual DD rhythms in *Pdf^01^* and *Pdfr* mutants occur in the evening, we propose that the LNd may contribute to the phase advanced LD evening behavior in these flies. Nonetheless, *disco* mutant flies that lack intact LNs but retain DNs also retain evening anticipation; this suggests redundant LN and DN pathways for evening behavior [44]. GLASS+ DN1s are missing in *glass* mutant flies and these flies display an intact evening peak but an altered morning peak, in that this peak is poorly entrained and variable in phase [45]. This suggests that the GLASS+ DN1 may be important for morning behavior. Additional functional cell-specific reagents will be necessary to assess the relative contribution of the PDF-sLNv, LNd, DN1, and DN3 in PDF-dependent circadian behaviors.

While our data suggest that the E cells are an important conduit for PDF action in the brain especially for circadian period, phase, and morning behavior, we also find that multiple targets are likely important for regulating rhythmic strength (Figure 6). In E cell only rescue, we do not observe significant rescue of rhythmic strength, indicating that other cells are relevant. Knockdown of pan-neuronal rescue in PDF neurons substantially reduces rhythmic strength (Table 2). On the other hand, *Pdf*GAL4-mediated rescue does not rescue DD rhythmicity. Thus, PDFR function in PDF neurons is necessary but not sufficient for DD rhythmic strength. Based on our expression analyses of *Pdfr*GAL4 (Figure 5) and PDF responsiveness by PDF application [28], these target cells are likely the PDF+ small LNv. We have observed a similar function for the LNv in regulating rhythmic strength in tissue-specific rescue of *na* mutants [16]. Desynchronized molecular rhythms in these cells may contribute to the reduction in rhythmic strength observed in *Pdf^01^* mutants [22].

Importantly, PDF neurons are not the only targets of PDF relevant to sustaining DD rhythms. Expression in broader sets of neurons including E cells (*cry*GAL4-13), most circadian pacemaker neurons (*clock*GAL4), and all neurons (*elav*GAL4) results in progressively increasing levels of rhythmicity (Table 2). In addition, *Pdf*GAL80 knockdown of pan-neuronal rescue does not suppress rhythmicity to mutant levels, further highlighting the role of both PDF neurons and non-PDF neurons in DD rhythmicity.

The rescue data and *Pdfr*GAL4 pattern presented here are also largely consistent with a report on PDF-responsiveness in the adult *Drosophila* brain [28]. Shafer et al. [28] observe PDF responsiveness in each of the circadian neuron groups (PDF+ sLNv, non-PDF sLNv, lLNv, LNd, DN1, DN2, DN3), albeit only weak responsiveness in a subset of lLNv assayed. The LN responsiveness matches *Pdfr*GAL4 quite well, as we observe *Pdfr*GAL4/UAS-GFP expression in all sLNv, all LNd, and weakly in a subset of lLNv (Figure 5B). Among the DN clusters, we observe *Pdfr*GAL4/UAS-nGFP in approximately half of the DN1 (Figure 5C), reproducibly in two DN3, and occasionally in one of the two DN2 (unpublished data). Whereas Shafer et al. report PDF responsiveness in most DN cells assayed, these experiments were performed using a *cry*GAL4-39/UAS-*Epac-cyclicAMP* reporter. *cry*GAL4-39 expression has been reported to include only a subset of DN1s and DN3s, and (in some reports) DN2s, comparable to the DN pattern we describe for *Pdfr*GAL4 [21],[28],[42]. Moreover, as noted above, these PDF-response measurements could reflect some degree of indirect responsiveness.

Despite the likely complexity of PDF function in circadian behavior, the data presented here define a major direct output pathway for PDF-dependent circadian behaviors. These studies highlight both the function in resetting core clocks as well as communicating timing information downstream of these core oscillators. It will be of interest to further refine the targets in the circadian system as well as define the molecular and cellular mechanisms by which PDF acts on those neural circuits to regulate circadian behavior.

## Materials and Methods

### Behavior Experiments and Analyses

For rescue experiments, either *Pdfr^han5304^* [18], *Pdfr^han5304^*;; UAS-*Pdfr* [20], or *Pdfr^han5304^*; *elav*GAL4 [46] virgin females were crossed to *y w*, GAL4/GAL80, or UAS males. For overexpression experiments, UAS-*Pdfr* (line 10) flies were crossed to either *y w* (control) or specific GAL4/GAL80 strains. For *Pdfr*GAL4 rescue experiments, female progeny were used for behavioral assays. For all other behavior, male progeny were assayed.

Locomotor activity levels were monitored using Trikinetics Activity Monitors for 5 d of LD followed by 7 d of DD at 25°C. For LD analyses (Figure 1), activity levels from each fly were normalized and averaged within genotypes over 4 d, as described previously [47]. For DD analyses (Figures 2 and 4), activity levels were normalized and averaged over the last 2 d of LD followed by 7 d of DD. To calculate time of evening anticipation in LD (Table 1), we determined the largest 2-h increase in normalized average activity for each fly over the last 7 h of the light phase. The time designation refers to the end point of the maximal activity increase, as averaged among individual flies in each genotype.

To quantitatively analyze morning behavior, we examined the first day of DD, as the lights-on peak in LD can mask the increase in morning behavior. To calculate DD Day 1 Morning Index (Table 1), normalized activity levels were averaged over three consecutive 30-min time points. For each genotype, maximum average activity of the group was determined for any two consecutive 30-min time points over the 6 h surrounding CT 0 (ZT 21- CT3). Minimum average activity was then determined for all time points before and after the observed maximum activity, up to 7 h before or after CT 0 (ZT 17- CT 7). Morning index value was obtained by subtracting the average of these minimum values from the maximum activity value.

For DD rhythmicity (Table 2), chi-squared periodogram analyses were performed using Clocklab (Actimetrics). Rhythmic flies were defined as those in which the chi-squared power was ≥10 above the significance line. Period calculations also considered all flies with rhythmic power ≥10, with the exception of one outlier removed as indicated. All *p*-values reported were calculated using Student's two-tailed *t*-tests.

### PERIOD Immunohistochemistry, Microscopy, and Quantification

Male *Pdfr^han5304^;UAS-Pdfr/+* and *UAS-Pdfr*/+ flies were entrained for 3–5 d at 25°C and anesthetized with CO_2_. The flies were dissected in 3.7% formaldehyde diluted in PBS at ZT1, ZT7, ZT12, and ZT18. After fixing for 30 min at room temperature, the brains were rinsed three times in PBS and incubated in PBT (PBS with 0.1% Triton) for 10 min at room temperature. The brains were then incubated with 5% goat serum diluted in PBT for 30 min at room temperature, followed by overnight incubation of 1∶4,000 rabbit anti-PER diluted in PBT containing 5% goat serum at 4°C. After several PBT rinses, the brains were incubated with 1∶500 goat-anti-rabbit AlexaFluor 594 (Amersham) in PBT overnight at 4°C. Final rinses in PBT and PBS were followed by mounting in 80% glycerol diluted in PBS. All slides were coded as to sample identity and remained so until the numerical analysis stage. PER-stained specimens were photographed with 60× oil lens on a Nikon Eclipse 800 laser scanning confocal microscope. For a given experiment the microscope, laser, and filter settings were held constant, and all specimens were photographed in the same microscopy session. PER immunostaining was quantified from digitally projected Z stacks using ImageJ (NIH). PER-stained soma were outlined to obtain average pixel intensity. On each projection image an unstained area was quantified to be used for background subtraction. All background-subtracted intensity measurements within a condition (time and genotype) were averaged. To combine experiments, background subtracted measurements were scaled to ZT1 of *Pdfr^han5304^;UAS-Pdfr/+* in that experiment. Statistical analysis was conducted in SPSS and Excel using ANOVA.

### PdfrGAL4

Targeted transposition was used to replace *P{EY11181}*, a P-element insertion approximately 40 bp upstream of the *Pdfr* transcription start site, with P{GawB}, a P-element containing GAL4. To perform targeted transposition, *P{EY11181}*, *P{GawB}* CyO flies were crossed to P-element transposase [29]. Strains in which *P{GawB}* mobilized to the X chromosome were identified by eye color and then analyzed by genomic PCR, to determine whether the GAL4 element had inserted into the *pdfr* upstream region. One strain (*Pdfr*GAL4-19) was identified using this method, and the insertion position of the GAL4 element was confirmed using inverse PCR (Model Systems Genomics, Duke University). For expression analyses, *Pdfr*GAL4 flies were crossed to UAS-nuclearGFP (UAS-nGFP). Female progeny were entrained, dissected, and labeled with anti-PER protein as previously described [16]. Images were obtained using laser scanning confocal microscopy (Nikon C1).

## Supporting Information

Figure S1 **Expression of PDFR using** ***clock*** **GAL4.** (A–E) Normalized activity plots for adult male populations, averaged over 4 d of LD entrainment. Light phase is indicated by white bars, whereas dark phase is indicated by black bars. Evening anticipation phase (ZT) is indicated below the genotype. Error bars represent standard error of the mean (*n* = 20–82). (A) UAS-*Pdfr*/+; (B) *Pdfr^han5304^*; UAS-*Pdfr*/+; (C) *Pdfr^han5304^*; UAS-*Pdfr*/*clock*GAL4; (D) *clock*GAL4/+; (E) *clock*GAL4/UAS-*Pdfr*.
(7.55 MB TIF)

## Abbreviations

DD: constant-dark conditions
E: evening
GFP: green fluorescent protein
LD: light-dark
M: morning
NA: narrow abdomen
PDF: pigment dispersing factor
PDFR: PDF receptor
PER: period
PI: pars intercerebralis
