# Supplementary figures and images for: The Neuropeptide PDF Acts Directly on Evening Pacemaker Neurons to Regulate Multiple Features of Circadian Behavior
Source: PLoS Biol. 2009 Jul 21;7(7):e1000154. doi: 10.1371/journal.pbio.1000154 (PMC2702683; doi:10.1371/journal.pbio.1000154)

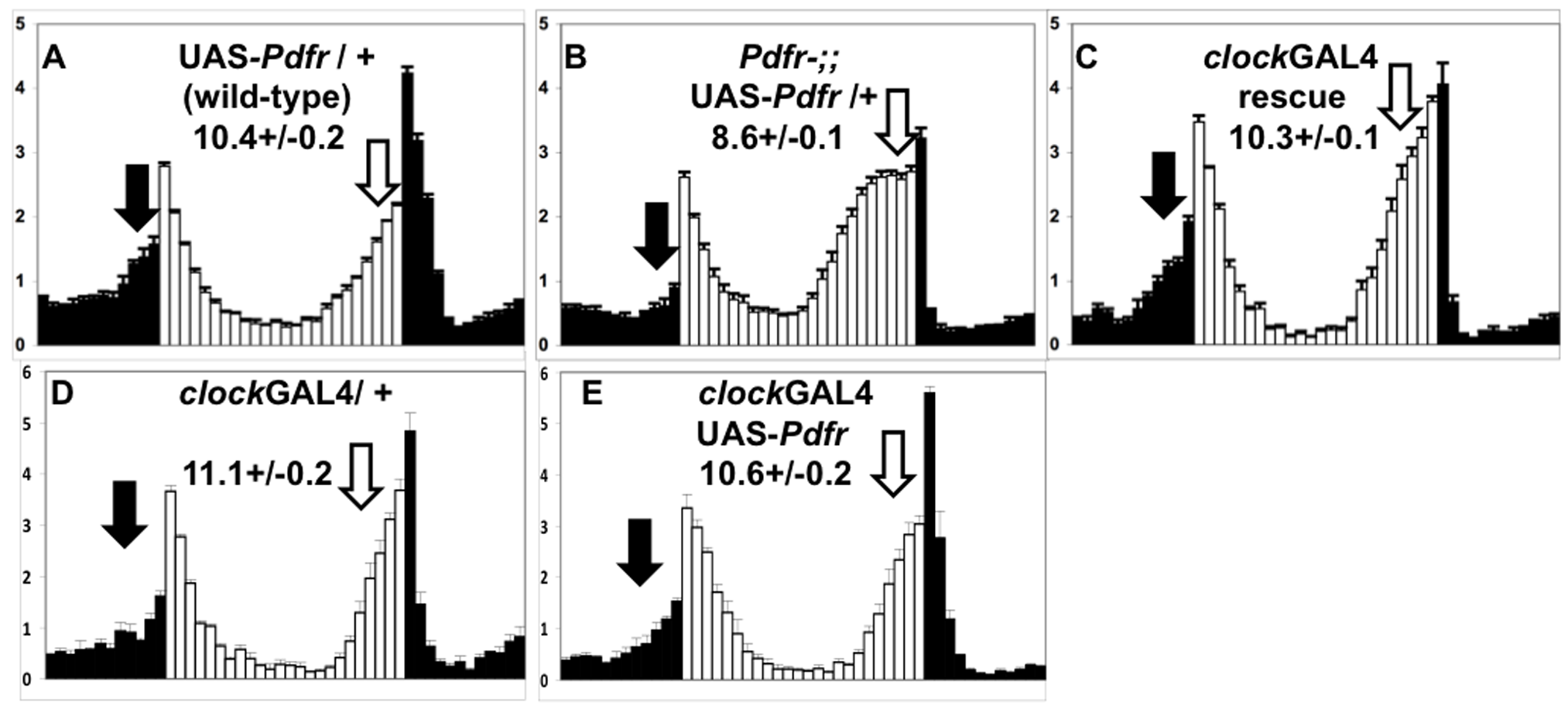

Supplement: Figure S1 — Expression of PDFR using clock GAL4. (A–E) Normalized activity plots for adult male populations, averaged over 4 d of LD entrainment. Light phase is indicated by white bars, whereas dark phase is indicated by black bars. Evening anticipation phase (ZT) is indicated below the genotype. Error bars represent standard error of the mean (n = 20–82). (A) UAS-Pdfr/+; (B) Pdfrhan5304; UAS-Pdfr/+; (C) Pdfrhan5304; UAS-Pdfr/clockGAL4; (D) clockGAL4/+; (E) clockGAL4/UAS-Pdfr. (7.55 MB TIF) [file pbio.1000154.s001.tif]
